# Effects of Autoinducer-2 on rumen bacterial community succession during in vitro fermentation with oat and *Suaeda salsa* straws

**DOI:** 10.3389/fmicb.2026.1880916

**Published:** 2026-08-05

**Authors:** Huiyun Liu, Xiaoxue Zhang, Chongyin Wang, Xiaozhen Liu

**Affiliations:** 1 Inner Mongolia Key Laboratory of Grassland Protection Ecology, Institute of Grassland Research, Chinese Academy of Agricultural Sciences, Hohhot, China; 2 College of Grassland Agriculture, Northwest A&F University, Yangling, Shaanxi, China

**Keywords:** Autoinducer-2, quorum sensing, rumen microbiota, serial passage, straw substrates

## Abstract

In order to reveal the effect of Autoinducer-2 (AI-2) quorum sensing signal on rumen bacterial community under roughage substrates with significant differences in chemical composition (oat straw has high lignocellulose and low crude protein, *Suaeda salsa* straw has higher crude protein and soluble carbohydrate content, and ash salt content). In this study, oat straw and *Suaeda salsa* straw were used as substrates, combined with *in vitro* batch culture fermentation and AI-2 addition treatment, the changes of bacterial community structure, interaction network, predicted metabolic function and xylanase activity were systematically analyzed. The community structures of the oat straw and *Suaeda salsa* systems differed significantly, and their disparities amplified across serial subcultures. Regulation of AI-2 on the communities showed obvious substrate dependence: in the oat system, AI-2 significantly reshaped the community structure and increased species diversity, whereas in the *Suaeda salsa* system, it showed a opposite trend. Network analysis showed that AI-2 treatment reduced community connectivity and increased the proportion of negative correlation edges, indicating that it could affect community stability by reconstructing competition and cooperation among species. Functional prediction and enzyme analysis revealed AI-2 enriched carbon metabolism pathways and raised xylanase activity across both substrates, with distinct regulated metabolic pathways observed in the two substrate cultures. This study provides a new perspective for understanding bacterial community succession regulated by quorum sensing signals in *in vitro* culture systems.

## 1 Introduction

Ruminants play a vital role in global agroecosystems and food supply chains by converting lignocellulosic biomass into high-value animal products such as meat and milk. The rumen is an efficient anaerobic reactor capable of degrading lignocellulose, driven mainly by bacteria (Ozsefil et al., 2024). The core driving force of this process is derived from the complex community of bacteria, fungi, protozoa and other microbial groups in the rumen. At the same time, these bacteria are in a dynamic equilibrium state and play an important role in the degradation of lignocellulosic biomass (Weimer, 2015). These rumen microorganisms synergistically decompose lignocellulose, hemicellulose, and cellulose in the straw matrix to convert them into volatile fatty acids (VFAs), CH_4_, and other products (Liang et al., 2020). Their community structure and function are closely related to feed digestion efficiency, host health, and environmental sustainability of ruminant breeding.

As a rich and low-cost agricultural by-product, crop straw is widely used as roughage in ruminant production. However, there are significant differences in the chemical composition of different straw substrates, and their lignin content, cellulose crystallinity and hemicellulose composition are different. These characteristics directly affect the colonization and metabolism of rumen microorganisms (Xu et al., 2019; Zhang et al., 2024). Previous studies have shown that the type of straw matrix is a key factor in shaping the rumen microbial community structure : for example, high-lignin straw enrich lignin-degrading microorganisms, while straw with high hemicellulose content tends to selectively enrich hemicellulose-degrading bacteria (Dao et al., 2021; Vahidi et al., 2021). However, most of the existing studies focus on the differences in microbial community composition induced by matrix types, and little is known about the internal regulatory mechanisms that drive these community changes. In particular, there is a lack of in-depth understanding of how exogenous addition of key signaling molecules regulates the dynamics of matrix-dependent microbial communities during continuous fermentation.

QS is a kind of intercellular communication mechanism mediated by small signal molecules called self-inducers (AIs), which enables bacteria to monitor population density and collectively coordinate gene expression (Neiditch et al., 2005; Papenfort and Bassler, 2016). This mechanism plays a key role in regulating a variety of microbial physiological processes, including biofilm formation, enzyme secretion, antibiotic production and pathogenic factor expression (Li et al., 2012; Mukherjee and Bassler, 2019). However, AI-2 can be recognized and produced by a variety of bacteria and plays a role in cross-species communication between Gram-positive and Gram-negative bacteria (Li J. et al., 2023). Recent studies have shown that AI-2 mediated quorum sensing is involved in regulating the assembly and function of microbial communities in ecosystems such as gut, soil and activated sludge (Thompson et al., 2015; Li J. et al., 2023). The regulatory role of AI-2 mediated quorum sensing in the rumen ecosystem is not yet clear. Especially under the condition of exogenous AI-2 addition, there is still a lack of systematic research on how to regulate the structure and function of rumen microbial community in different straw substrates during continuous fermentation.

This study aimed to explore the effects of straw type and AI-2 on rumen bacterial community dynamics during serial batch subculture, addressing three questions: (1) How do substrate type and AI-2 affect bacterial community succession? (2) What are the effects of AI-2 on bacterial community structure, diversity, and functional potential? (3) Which key bacterial taxa respond to AI-2 regulation?

## 2 Materials and methods

### 2.1 Experimental sample treatment and subculture

#### 2.1.1 Ruminal fluid sample collection and pretreatment

Fresh rumen contents were collected from healthy adult sheep in slaughterhouses near Hohhot. Immediately after slaughter, the rumen contents were collected aseptically, sealed in a sterile anaerobic bag, and quickly transported back to the laboratory. In the process of sample collection, transportation and pretreatment, the operation time should be shortened as much as possible to reduce the effect of oxygen exposure on rumen microbial activity.

The collected rumen contents were fully mixed and filtered with 4 layers of sterile gauze to remove large pellet feed residues and impurities. The filtrate was used as rumen fluid for the experiment. The filtered rumen fluid was stored at 4 °C and inoculated into an *in vitro* fermentation system containing different straw substrates according to the experimental design for continuous subculture and subsequent microbial community structure analysis.

#### 2.1.2 Straw substrate pretreatment

In this experiment, two straw substrate treatment groups were set up, namely oat straw and *Suaeda salsa* straw. The straw samples were dried to constant weight at 65 °C, crushed and transferred to sterile anaerobic bottles. The straw samples were sterilized by high-pressure steam sterilization (121 °C, 30 min) to eliminate the interference of miscellaneous bacteria. After sterilization, the straw samples were cooled to room temperature on an ultra-clean bench, sealed and stored at 4 °C.

#### 2.1.3 Construction and grouping of anaerobic culture system

In this study, inorganic salt medium was used as the basic anaerobic medium to construct an *in vitro* anaerobic system of rumen microorganisms. The final volume of the system was 60 mL, containing 0.6 g sterilized straw substrate. At the initial culture, the pretreated rumen fluid was added at a 10% (v/v, 6 mL) inoculation amount, and all operations were performed in an anaerobic chamber.

The experiment used a two-factor grouping design of straw type and AI-2 addition or not, a total of 4 treatment groups, and 5 biological replicates were set in each group to ensure the reliability of the results. The specific composition of each treatment group was as follows: In control group 1, oat straw was used as substrate, 6 mL rumen fluid was inoculated and equal volume of sterile water was added. Treatment group 1 used oat straw as substrate, inoculated rumen fluid and added 6 mL 100 μmol/L AI-2 reagent. In control group 2, 6 mL rumen fluid was inoculated with *Suaeda salsa* straw as substrate, and equal volume of sterile water was added. Treatment group 2 used *Suaeda salsa* straw as substrate, inoculated rumen fluid and added 6 mL 100 μmol/L AI-2 reagent.

#### 2.1.4 Serial passaging culture

The initial culture lasted for 6 days. After the end of the culture, the fermentation broth of each treatment group was absorbed according to 10% (v/v) inoculation amount, and then transferred to the freshly prepared corresponding medium. The above anaerobic culture conditions were repeated to complete the second generation culture. By analogy, continuous subculture to the fifth generation. After the end of each generation of culture, 30 mL of fermentation broth was taken from each replicate group and centrifuged at 4 °C and 6000 rpm for 20 min. The supernatant was discarded, the microbial precipitate was collected and stored in an ultra-low temperature refrigerator at −80 °C for subsequent 16S rRNA gene sequencing.

After the subculture was completed, the samples of the 0th, 1st, 3rd and 5th generations were selected for 16S rRNA gene sequencing analysis. Among them, A is the original rumen fluid sample of the 0th generation; the first generation samples included oat control group (Byck), oat AI-2 treatment group (ByAI_2), *Suaeda salsa* control group (Bjck) and *Suaeda salsa* AI-2 treatment group (BjAI_2). The third generation samples included oat control group (Cyck), oat AI-2 treatment group (CyAI_2), *Suaeda salsa* control group (Cjck) and *Suaeda salsa* AI-2 treatment group (CjAI_2). The fifth generation of samples included oat control group (Dyck), oat AI-2 treatment group (DyAI_2), *Suaeda salsa* control group (Djck) and *Suaeda salsa* AI-2 treatment group (DjAI_2) (Supplementary Data Sheet 1).

### 2.2 DNA extraction

For the pretreated samples, the MagBeads FastDNA Kit for Soil (116564384) (MP Biomedicals, CA, USA) kit was used to extract nucleic acids. The extracted DNA was subjected to 0.8% agarose gel electrophoresis to determine the molecular size, and the DNA was quantified by Nanodrop.

### 2.3 16 S rRNA gene amplicon sequencing

PCR amplification of the bacterial 16S rRNA genes V3–V4 region was performed using the forward primer 338F (5′-ACTCCTACGGGAGGCAGCA-3′) and the reverse primer 806R (5′-GGACTACHVGGGTWTCTAAT-3′). Sample-specific 7-bp barcodes were incorporated into the primers for multiplex sequencing. The PCR components contained 5 μL of buffer (5×), 0.25 μL of Fast pfu DNA Polymerase (5U/μL), 2 μL (2.5 mM) of dNTPs, 1 μL (10 uM) of each Forward and Reverse primer, 1 μL of DNA Template, and 14.75 μL of ddH_2_O. Thermal cycling consisted of initial denaturation at 98 °C for 5 min, followed by 26 cycles consisting of denaturation at 98 °C for 30 s, annealing at 52 °C for 30 s, and extension at 72 °C for 45 s, with a final extension of 5 min at 72 °C. PCR amplicons were purified with Vazyme VAHTSTM DNA Clean Beads and quantified using the Quant-iT PicoGreen dsDNA Assay Kit (Invitrogen, Carlsbad, CA, USA). After the individual quantification step, amplicons were pooled in equal amounts, and pair-end 2 × 250 bp sequencing was performed using the Illlumina NovaSeq platform with NovaSeq 6000 SP Reagent Kit (500 cycles) at Shanghai Personal Biotechnology Co., Ltd.

### 2.4 Sequence analysis

Microbiome bioinformatics were performed with QIIME2 2022.11 (Bolyen et al., 2018) with slight modification according to the official tutorials^1^. Briefly, raw sequence data were demultiplexed using the demux plugin following by primers cutting with cutadapt plugin (Martin, 2011). Sequences were then quality filtered, denoised, merged and chimera removed using the DADA2 plugin (Callahan et al., 2016). Non-singleton amplicon sequence variants (ASVs) were aligned with mafft (Katoh et al., 2002) and used to construct a phylogeny with fasttree2 (Price et al., 2009). Beta diversity metrics weighted UniFrac, unweighted UniFrac (Lozupone and Knight, 2005), Jaccard distance, and Bray-Curtis dissimilarity) were estimated using the diversity plugin with samples were rarefied to 33052 sequences per sample. Taxonomy was assigned to ASVs using the classify-sklearn naive Bayes taxonomy classifier in feature-classifier plugin (Bokulich et al., 2018) against the Greengenes2(2022. 10) Database.

### 2.5 Bioinformatics and statistical analysis

Sequence data analyses were mainly performed using QIIME2 and R packages (v3.6.0). ASV-level ranked abundance curves were generated to compare the richness and evenness of ASVs among samples. Beta diversity analysis was visualized via principal coordinate analysis (PCA). The significance of differentiation of microbiota structure among groups was assessed by PERMANOVA (Permutational multivariate analysis of variance) (McArdle and Anderson, 2001), ANOSIM (Analysis of similarities) (Clarke, 1993; Warton et al., 2012), Permdisp (Anderson, 2006) using QIIME2. The taxonomy compositions and abundances were visualized using MEGAN (Huson et al., 2011) and GraPhlAn (Asnicar et al., 2015). Taxa abundances at the ASV levels were statistically compared among samples or groups by MetagenomeSeq (Zgadzaj et al., 2016). LEfSe (Linear discriminant analysis effect size) was performed to detect difierentially abundant taxa across groups using the default parameters (Segata et al., 2011). OPLS-DA (Orthogonal Partial Least Squares Discriminant Analysis) was also introduced as a supervised model to reveal the microbiota variation among groups, using the R package “muma” (Mahadevan et al., 2008). Random forest analysis was applied to discriminating the samples from different groups using QIIME2 with default settings (Breiman, 2001; Liaw and Wiener, 2007). Nested stratified k-fold cross validation was used for automated hyperparameter optimization and sample prediction. The number of k-fold cross-validations was set to 5. Co-occurrence network analysis was performed by SparCC analysis. The pseudocount value in SparCC was set to 10^–6^. The cutoff of correlation coefficients was determined as 0.7 through random matrix theory-based methods as implemented in R package RMThreshold. Based on the correlation coefficients, we constructed co-occurrence network with nodes representing ASVs and edges representing correlations between these ASVs. Network was visualized using R package igraph and ggraph. Microbial functions were predicted by PICRUSt2 (Phylogenetic investigation of communities by reconstruction of unobserved states) (Douglas et al., 2020) upon MetaCyc^2^ and KEGG^3^ databas. Xylanase activity was determined using a commercial xylanase assay kit following the manufacturer’s protocols.

## 3 Results

### 3.1 The regulation of AI-2 on xylanase activity under different substrate conditions

**FIGURE 1:**
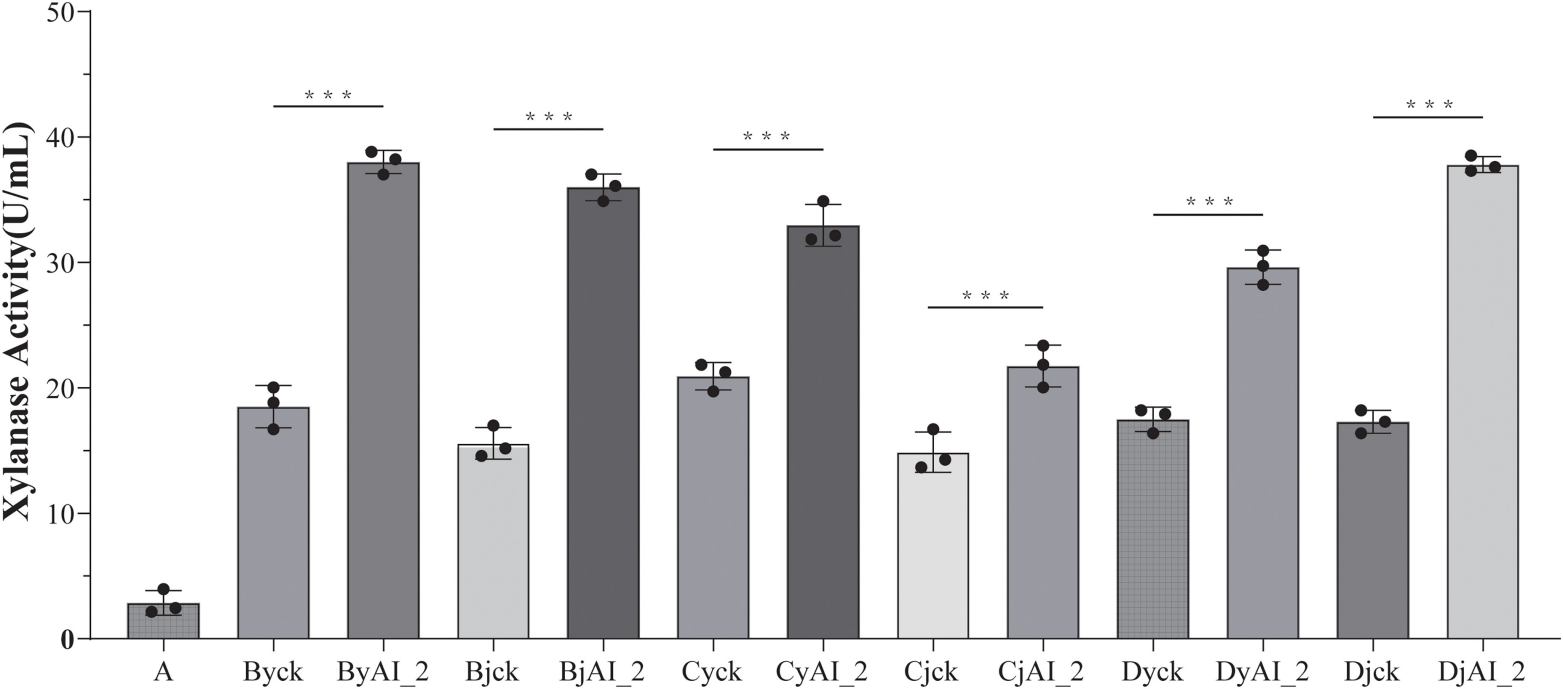
The changes of xylanase activity under different substrates and AI-2 treatment. Bars represent mean ± SD. Statistical significance is indicated as *P* < 0.001(***).

In the fermentation system with oats and *Suaeda salsa* as substrates, the xylanase activity was significantly higher in the AI-2-supplemented groups than in the corresponding control groups, confirming that the *in vitro* system supported active microbial fermentation (Figure 1).

### 3.2 Analysis of microorganism community diversity

**FIGURE 2:**
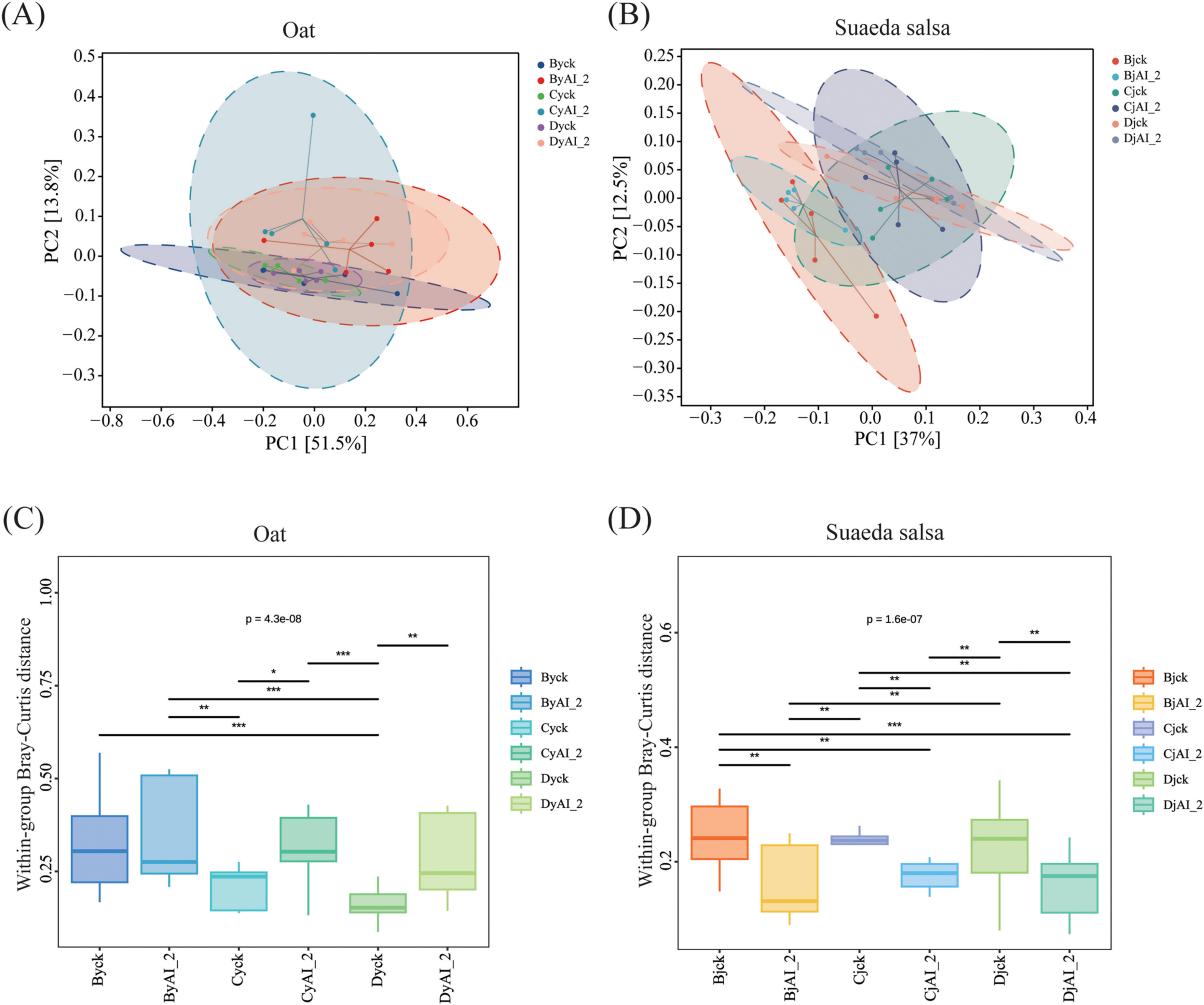
PCoA analysis and boxplot display of microbial community composition under Oat and *Suaeda salsa* substrates in different treatment groups. **(A)** PCoA analysis of microbial communities under different treatments on oat substrate; **(B)** PCoA analysis of microbial communities under different treatments on *Suaeda salsa* substrate; **(C)** boxplot showing microbial community diversity under different treatments on oat substrate; **(D)** boxplot showing microbial community diversity under different treatments on *Suaeda salsa* substrate. The boxplot represents the mean ± SD. Statistical significance is indicated as *P* < 0.05(*), *P* < 0.01(**) and *P* < 0.001(***).

The results of PCA analysis showed that PC1 and PC2 in the oat substrate group explained 65.3% of the bacterial variation, and partial compositional separation existed between the control group and the AI-2 treatment group along the PC1 axis. The two axes of *Suaeda salsa* substrate group only explained 49.5% of the variation. Serial subcultures reduced the overlap of the confidence ellipse between the control group and the treatment group, and the regulatory effect of AI-2 on microbial communities was reinforced during successive cultivation (Figures 2A,B).

The analysis of Bray-Curtis distance within the group further showed that there were also significant substrate differences in the regulation of AI-2 on community homogeneity: after adding AI-2 to oat substrate, the distance within the third and fifth generations increased, and the dispersion of community composition within the group increased. After the addition of AI-2 to the *Suaeda salsa* substrate, the distance within the group decreased, and the community structure of the replicated samples in the group became more homogeneous (Figures 2C,D).

### 3.3 Phylogenetic relationship of microbial core species

**FIGURE 3:**
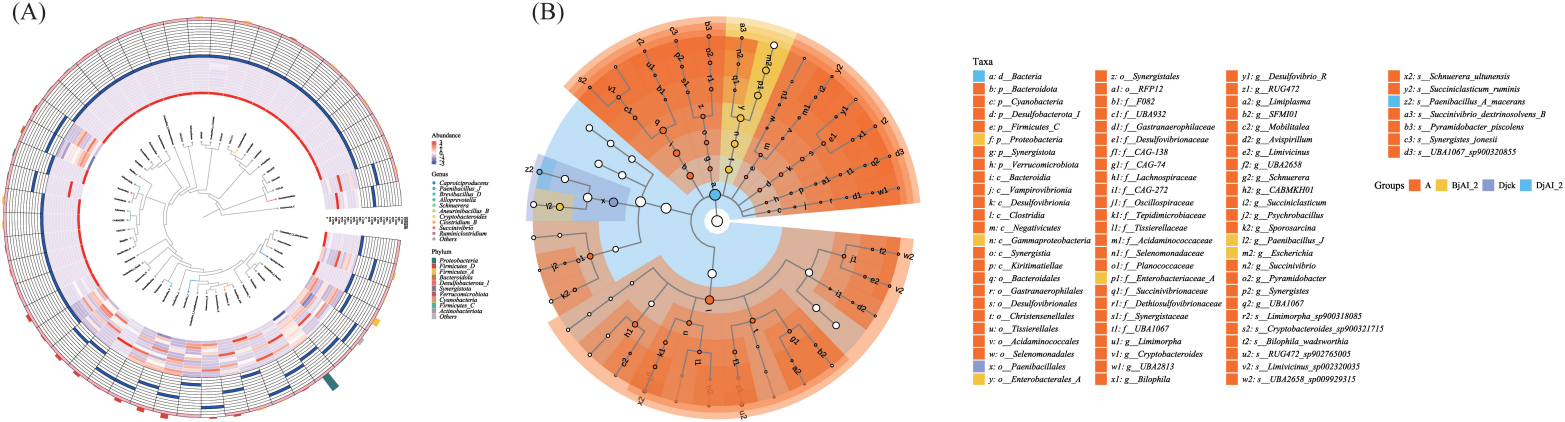
Phylogenetic distribution and differential enrichment of key bacterial taxa across treatments and generations. **(A)** The circular phylogenetic tree illustrates the evolutionary relationships among major taxonomic units; the outer ring displays a relative abundance heatmap, where varying shades indicate the abundance levels of corresponding taxonomic units across samples and treatment groups. The lateral legend provides taxonomic annotations. **(B)** The LEfSe differential analysis phylogenetic tree displays taxonomic units significantly enriched across different treatment groups. Dots represent various taxonomic levels from phylum to genus/species; dot color indicates the primary treatment group where the unit is enriched, while dot size correlates with the effect size of the differential effect. The right-hand list provides names of significantly differentially enriched microbial communities.

At the phylum level, the bacterial community was dominated by Firmicutes and Proteobacteria. Meanwhile, significant structural differentiation was observed among samples and treatment groups, suggesting that substrate type, serial subculture, and AI-2 intervention jointly drive the reshaping of bacterial communities. Further, from a more detailed classification level, some key genera showed significant abundance fluctuations among samples. For example, *Paenibacillus* marked in the phylogenetic tree showed differential enrichment under different conditions, indicating that these bacteria are more sensitive to environmental changes (Figure 3A).

The results of LEfSe analysis showed that the community diversity and total abundance of group A were the highest, and the core dominant flora belonged to Bacteroidota. In contrast, the BjAI_2 group enriched facultative anaerobic bacteria *Paenibacillus* and *Escherichia*, and the two types of microorganisms could proliferate in the *in vitro* culture system. The dominant species in the DjAI_2 group was *Paenibacillus_A macerans*, which had both the ability of compound carbon source degradation and environmental stress resistance. In the fifth generation system of *Suaeda salsa* substrate, AI-2 treatment enriched the polysaccharide-utilizing stress-resistant bacteria, which ensured the metabolic activity and ecological stability of the community during continuous passage (Figure 3B).

### 3.4 AI-2-mediated identification of core differential species

**FIGURE 4:**
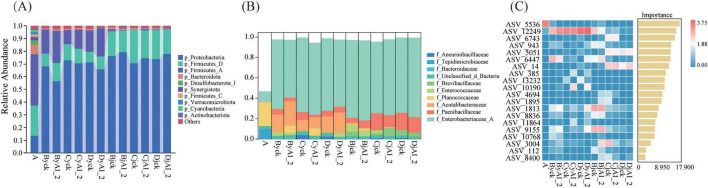
Microbial community composition and feature importance across different treatments and groups. **(A)** Relative abundance at the phylum level of microorganisms across different treatment groups; **(B)** Stacked bar chart of species contribution at the family level among different treatment groups; **(C)** ASV feature importance in random forest analysis.

After the introduction of oat and *Suaeda salsa* substrate and the application of AI-2 treatment, the microbial community structure was significantly reshaped. The relative abundance of Proteobacteria in all samples increased significantly and gradually dominated. On the contrary, the overall abundance of Firmicutes decreased from 63% to 25% after the introduction of oat and *Suaeda salsa* substrates and the application of AI-2, but different subgroups in Firmicutes showed significant differential responses. Among them, the relative abundance of Firmicutes_A decreased overall, but this change was regulated by the interaction effect of AI-2 and substrate type: in the oat system, AI-2 significantly promoted the enrichment of Firmicutes_A, while in the *Suaeda salsa* system, AI-2 reduced the abundance of Firmicutes_D. The above results indicate that the regulation effect of AI-2 on microbial community has obvious substrate dependence (Figure 4A).

In the analysis of the species contribution of the microbial community at the family level, Enterobacteriaceae_A was the absolute dominant group in all samples, and its relative abundance was dominant in each group, which constituted the core skeleton of the rumen fermentation microbial community. Specifically, in the oat substrate group, the contribution of the Acutalibacteraceae family was relatively high; in the *Suaeda salsa* substrate group, the abundance of Paenibacillaceae was more prominent. In the oat group, AI-2 significantly enhanced the contribution of Acutalibacteraceae family. In the *Suaeda salsa* group, AI-2 significantly increased the abundance ratio of Enterobacteriaceae_A (Figure 4B).

A few ASVs such as ASV_5536, ASV_12249, ASV_6743 and ASV_943 showed extremely high characteristic importance and were the core biomarkers to distinguish the community structure of each group. The abundance distribution of these key ASVs has significant substrate specificity. Among them, ASV_12249, belonging to Acutalibacteraceae, was significantly enriched in oat and AI-2 treatment groups, while the abundance in *Suaeda salsa* group was low. In contrast, ASV_943 and ASV_6447 belonging to the Enterococcaceae family, ASV_14 belonging to the *Paenibacillus* family, and unclassified ASV_5051 showed higher abundance in the *Suaeda salsa* group. The above results indicate that different substrate conditions can selectively enrich the dominant microbiota at different family levels and form a clear substrate response pattern. These key ASVs are important indicator microbiotas that drive community differentiation and substrate adaptive response (Figure 4C).

### 3.5 The core mechanism of AI-2 mediating the difference of microbial regulation function

**FIGURE 5:**
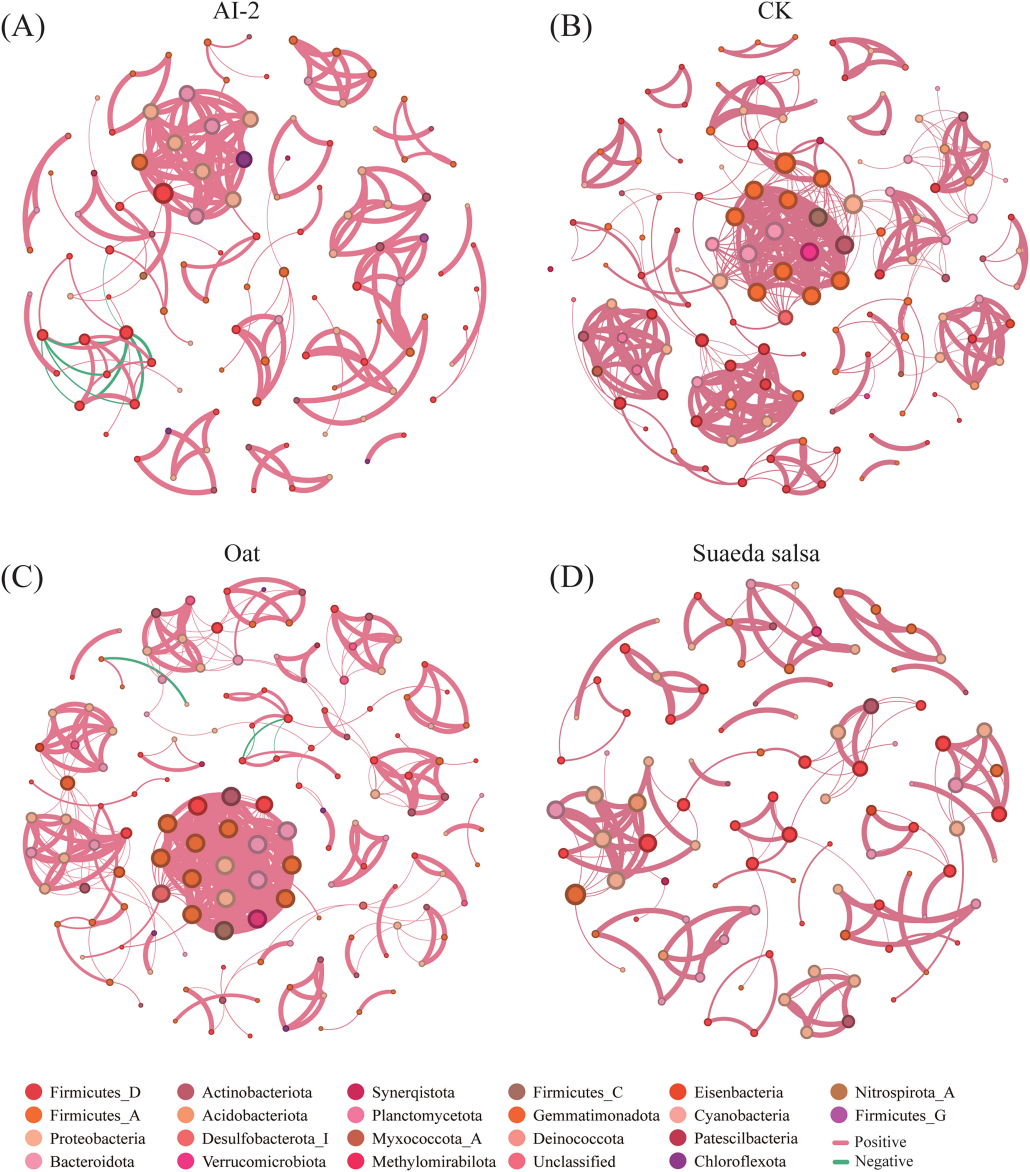
Comparison of microbial co-occurrence networks under different treatments and substrates. **(A)** AI-2 treatment; **(B)** control (CK); **(C)** oat straw substrate; **(D)** *Suaeda salsa* straw substrate. Nodes represent microbial taxa. Node size indicates taxon importance in the network, and node color denotes phylum-level taxonomy (see legend). The line represents the correlation between different classification units, pink represents positive correlation, and green represents negative correlation.

Co-occurrence network analysis showed that AI-2 treatment significantly changed the microbial interaction pattern: compared with the control group, the network connection of AI-2 group was looser and the negative correlation edges increased, suggesting that AI-2 regulated resource allocation and niche competition through quorum sensing, enhance the negative correlation between species and promote community screening and structural reconstruction. In this context, Firmicutes_D and Proteobacteria are more likely to dominate. On the contrary, the network of the control group was closer and dominated by positive correlation, reflecting that the community interaction was more synergistic and the structure was more stable. It is worth noting that the oat substrate system showed a significant negative correlation even without AI-2, especially between Firmicutes and Proteobacteria, indicating that the substrate itself can induce competition and screening effects. The substrate system network of *Suaeda salsa* is dominated by positive correlation, suggesting that it is more inclined to maintain synergistic interaction. In general, the regulation of AI-2 on community interaction has a significant substrate background dependence: it is easier to strengthen competitive reconstruction in the oat system, while the synergistic structure is relatively more stable in the *Suaeda salsa* system (Figure 5).

### 3.6 Differences in functional pathways related to straw degradation regulated by AI-2

**FIGURE 6:**
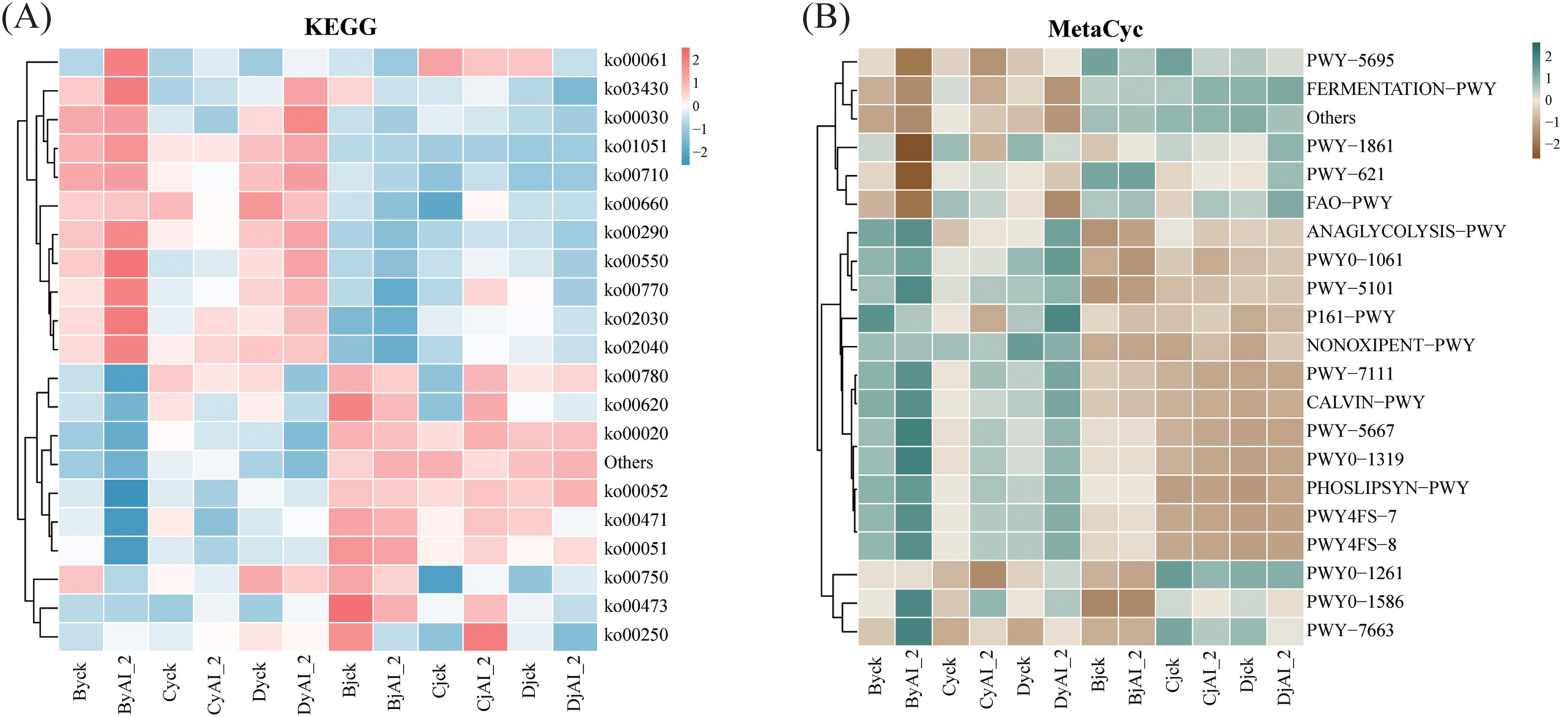
Prediction of microbial function under different substrates and AI-2 treatment. **(A)** Heatmap of KEGG functional pathway abundance across different treatment groups; **(B)** Heatmap of MetaCyc metabolic pathway abundance across different treatment groups.

The results of KEGG function prediction showed that different substrates and AI-2 treatment jointly drove the significant functional differentiation of the fermentation system. In the oat-based system, genes such as ko00061 and ko00030 were significantly enriched, and further enhanced after AI-2 treatment, suggesting that AI-2 promoted carbon flow redistribution and energy and reducing power supply through quorum sensing, thereby enhancing metabolic adaptation and biosynthesis needs under cellulose-based substrate conditions; in contrast, when *Suaeda salsa* was used as the substrate, the community functional spectrum was significantly shifted to the increase of glucose metabolism genes such as ko00051 and ko00052, indicating that microorganisms were more dependent on specific monosaccharide metabolism pathways in response to the *Suaeda salsa* substrates (Figure 6A).

The comprehensive MetaCyc functional pathway analysis showed that the microbial community showed significant metabolic strategy differentiation under different substrate backgrounds and was further regulated by AI-2. In the oat substrate system, the overall activity of PWY-5101, PWY-7111, CALVIN-PWY and PHOSLIPSYN-PWY pathways was higher, and further enhanced after AI-2 treatment, mainly pointing to amino acid synthesis, carbon flow rearrangement and assimilation, and membrane phospholipid biosynthesis, suggesting that oats as a cellulose substrate is more likely to drive the community to invest carbon sources into biomass construction and structural metabolism, thereby improving growth and adaptability. In contrast, FERMENTATION-PWY (fermentation pathway set) and FAO-PWY (fatty acid β-oxidation) and other pathways related to energy acquisition and substrate degradation were more active in *Suaeda salsa* substrate system, while PHOSLIPSYN-PWY, PWY-5101 and other anabolism and structural metabolism pathways were relatively weak, indicating that the community was more inclined to adopt the metabolic model of energy priority under the condition of *Suaeda salsa* as substrate (Figure 6B).

## 4 Discussion

Substrate chemical properties are important drivers shaping rumen bacterial community assembly (Zheng et al., 2021; Zhu et al., 2025). The oat straw used herein is rich in cellulose and hemicellulose with a homogeneous fibrous matrix, which may favor the proliferation of fibrolytic bacteria (Peterková et al., 2025). By contrast, the halophyte *Suaeda salsa* accumulates abundant soluble carbohydrates, mineral ash and secondary metabolites; these chemical traits impose distinct salt and metabolic stress that filters microbial populations (Zhao et al., 2011; Yuan et al., 2016). Consistent with these substrate disparities, bacterial community profiles diverged sharply between the two roughage groups in our fermentation system. This observation aligns with previous *in vitro* roughage incubation work by Pitta et al. (2016) and suggests substrate physicochemical traits shaped rumen bacterial assemblages across multiple taxonomic levels (Pitta et al., 2016). To minimize variation originating from the initial rumen inoculum and amplify sustained selective pressure exerted by substrate type, we implemented five consecutive generations of serial batch subculture. This serial passaging progressively increased community dissimilarities across experimental treatments, enabling robust characterization of bacterial adaptive responses under stabilized *in vitro* fermentative conditions (Elena and Lenski, 2003). One key limitation of this long-term serial culture system should be noted: successive transfers selectively reduce the relative abundance of certain obligate anaerobes and enrich taxa better adapted to *in vitro* subculture conditions, which shifts the composition of the *in vitro* microbial community away from the native *in vivo* rumen microbiota.

Autoinducer-2, as a widespread inter-specific and intra-specific quorum sensing signal, can participate in key processes such as bacterial colonization, metabolic division and community stability (Papenfort and Bassler, 2016), and quorum sensing itself is also an important regulatory mechanism for microorganisms to optimize resource utilization (Peng et al., 2025). The core finding of this study is that the regulatory effect of AI-2 on rumen bacterial communities exhibits significant substrate dependence: in the oat straw system, AI-2 significantly altered community structure and increased diversity, whereas in the *Suaeda salsa* system, it showed a weakened positive effect and even an inhibitory tendency. One possible explanation for this difference is that substrate type mediates distinct bacterial competitive and stress backgrounds: oat straw is a cellulose-rich substrate, where bacteria may face intense competition for carbon sources. AI-2 may promote functional complementarity among bacteria by regulating niche differentiation, thereby enhancing the community’s ability to synergistically utilize fibrous substrates and ultimately increasing bacterial diversity (Liu et al., 2022). In contrast, the *Suaeda salsa* system is characterized by high salt and ash contents, and microbial community assembly in this system may be more strongly shaped by stress tolerance and cooperative metabolic adaptation. These stress-associated conditions may reduce bacterial responsiveness to AI-2 signals and thereby limit its positive regulatory effect (Cornforth and Foster, 2013; Torres et al., 2019). These findings provide an important prerequisite for the subsequent use of quorum sensing to regulate rumen fermentation, and it is necessary to optimize the regulation strategy in combination with the type of roughage.

Firmicutes and Proteobacteria were the dominant phyla in this *in vitro* culture system, rather than the typical Firmicutes and Bacteroidetes-dominated structure commonly observed in the rumen (Sanjorjo et al., 2023; da Silva Pereira et al., 2025). This shift likely reflects community succession during *in vitro* serial subculture rather than the native rumen microbiota. This study shows that AI-2 can significantly regulate bacterial community composition and functional bacterial distribution in a substrate-dependent manner. Based on LEfSe analysis, group A showed the highest diversity and total abundance, and the enriched taxa were mainly associated with anaerobic fermentation, carbohydrate utilization, and metabolic intermediate conversion. Among them, the enrichment of *Mobilitalea*, *Cryptobacteroides* and *Succinivibrio* suggested that this group had strong potential of organic substrate fermentation and intermediate metabolite production (Taga and Xavier, 2011). The enrichment of *Succiniclasticum_ruminis* suggested a potential advantages in the further conversion of succinate to propionate (van Gylswyk, 1995), while *Schnuerera ultunensis* was involved in organic acid conversion under anaerobic conditions (Manzoor et al., 2018). Combined with the importance screening of random forest features, a batch of substrate-specific ASVs were further identified. These core markers were mainly Firmicutes (65%), especially Firmicutes_D subphylum. *Paenibacillus* and *Enterococcus* related ASVs accounted for 45%, which were the key groups in response to substrate and AI-2 treatment. The top three ASV features showed obvious substrate preferences: ASV_12249 was assigned to the genus *Caproiciproducens*, and its enrichment in the AI-2-treated oat straw group increased with serial passage. The bacteria are capable of secreting hemicellulase, which may partially account for the elevated xylanase activity observed in the oat substrate system. (Marichamy and Mattiasson, 2005; Liu et al., 2023). ASV_6743 belongs to *Paenibacillus_J*, maintains high relative abundance across all *Suaeda salsa* groups; this taxon may simultaneously possess polysaccharide-degrading capacity and salt tolerance traits. (Xi et al., 2022; Tang et al., 2023).

The early BjAI_2 group enriched facultative anaerobic *Paenibacillus* and *Escherichia coli*, and preferred to use soluble and easily fermentable carbon sources (Li G. et al., 2023). AI-2 in DjAI_2 group enriched *Paenibacillus_A macerans*, and relied on this kind of flora with both degradation and stress resistance to maintain the metabolic stability of the community during passage (Pason et al., 2006). According to the response of *Paenibacillus* to AI-2, two potential mechanisms can be proposed: one is the direct perception of the strain: First, some *Paenibacillus*-related taxa may directly respond to AI-2-associated quorum sensing signals. (Qian et al., 2015). Previous studies have reported that some aerobic endospore-forming bacteria can produce AI-2 and that LuxS/AI-2-related quorum sensing may regulate physiological traits such as biofilm formation and stress adaptation in related bacteria. (Duanis-Assaf et al., 2016) Such potential responsiveness may help these taxa improve carbon utilization and stress adaptation under specific substrate conditions. (Xi et al., 2022). The second is indirect community screening: AI-2 aggravates interspecific competition and expands the negative correlation of flora (Liu et al., 2022). The stress sensitive rumen bacteria are inhibited, and *Paenibacillus* with both polysaccharide degradation and stress tolerance has a survival advantage (Mizrahi et al., 2021; Chen et al., 2023). The increase in abundance is an indirect result of flora reconstruction. Under the conditions of this experiment, two mechanisms may coexist: under the condition of oat straw substrate, although the fibrous carbon source is abundant, its structure is complex and its availability is limited, which may promote the competition and differentiation of bacteria for available degradation products and niches. AI-2 correlates with the enrichment of *Caproiciproducens* (ASV_12249) and elevated xylanase activity across both substrates. In the *Suaeda salsa* system specifically, AI-2 may filter stress-tolerant *Paenibacillus* taxa by reshaping interspecies interactions, which could sustain community metabolic homeostasis throughout successive transfers. In addition, this study also detected three highly important unclassified ASVs, accounting for 15% of the total key markers, indicating that there are still a large number of unannotated functional microorganisms in the system, which need to be further isolated and verified and developed into unconventional roughage fermentation agents.

Bacterial co-occurrence network analysis is a common analytical approach to characterize potential interspecific associations within microbial communities, which helps infer the synergistic or competitive relationships among taxa and evaluate community stability patterns (Barberán et al., 2012). This study found that AI-2 treatment reduced network connectivity and increased the proportion of negative correlations, promoting community reconstruction toward a more competitive structure. This is consistent with the view that quorum sensing maintains community balance by regulating niche competition (Su et al., 2023; Li et al., 2025). Notably, the oat substrate itself was associated with a higher proportion of negative correlations, suggesting stronger potential competition or niche differentiation. The *Suaeda salsa* system was dominated by positive correlations, reflecting an adaptive strategy of bacteria under stress, again confirming the decisive role of substrate properties in shaping bacterial interaction patterns (Qian et al., 2022). In the oat straw system, AI-2 treatment was associated with higher predicted potential for fatty acid biosynthesis, the pentose phosphate pathway and phospholipid biosynthesis, which may reflect enhanced carbon redistribution and biosynthetic demand during community succession. In the *Suaeda salsa* system, the predicted functions were mainly associated with monosaccharide metabolism and fermentation pathways, suggesting an energy-oriented adaptive strategy under substrate-associated stress. To further verify whether such AI-2-induced metabolic discrepancies at the community level were associated with the degradation of fibrous straw substrates, we determined the xylanase activity of each treatment group. Xylanase is a key rate-limiting enzyme for hemicellulose degradation. Our results showed that AI-2 significantly increased xylanase activity under both substrates, indicating that quorum sensing can enhance the degradation of straw cell-wall polysaccharides by regulating bacterial composition and functional gene expression (Mao et al., 2025). Despite the consistent promotion of xylanase activity by AI-2 across both substrates, the full causal pathway underlying substrate-dependent bacterial responses and enhanced hemicellulase activity still lacks direct molecular verification. The present study only provides correlative evidence including taxonomic biomarkers, predicted functional profiles and extracellular enzyme phenotypes, without transcriptomic or inhibitor-based assays to dissect the linear signal cascade from AI-2 recognition to xylanase gene transcription. Further targeted omics sequencing of dominant isolated strains and QS inhibitor intervention trials are required to eliminate indirect community interference and validate the core mechanistic chain in subsequent research. Together, these findings provide preliminary evidence that AI-2 may participate in regulating bacterial community succession and hemicellulose degradation during unconventional roughage fermentation.

## 5 Conclusion

This study systematically explored the regulation of exogenous quorum sensing signal molecule AI-2 on rumen bacterial community structure, potential microbial associations, functional metabolic pathways, and xylanase activity during *in vitro* serial batch subculture, and confirmed its significant substrate-dependent effects supported by multiple quantitative analyses of microbiota and phenotypic indicators. The results showed that AI-2 could affect the adaptation and utilization of rumen bacteria to different plant-derived fibrous substrates by reshaping community composition, altering bacterial interaction patterns, and driving differentiation in metabolic functions. In the oat straw system, AI-2 was more inclined to promote the enrichment of bacteria related to carbohydrate fermentation, complex substrate utilization and metabolic intermediate conversion, and promote the reconstruction of community to competitive interaction structure, which was verified by the significant enrichment of characteristic fibrolytic ASVs screened via random forest. In the *Suaeda salsa* straw system, AI-2 facilitated the enrichment of bacteria with both polysaccharide degradation potential and environmental adaptability represented by stress-resistant *Paenibacillus* biomarker ASVs with statistically higher abundance in AI-2 groups, thereby helping maintain metabolic activity and stability of the bacterial community during serial subculture. Functional prediction further indicated that AI-2 and substrate type jointly drove divergent metabolic strategies: the oat system favored carbon redistribution and biosynthetic metabolism, whereas the *Suaeda salsa* system was dominated by monosaccharide metabolism and fermentative energy production targeted at coping with high-salt substrate stress. Meanwhile, AI-2 significantly increased xylanase activity under both substrates, indicating a positive effect on hemicellulose degradation and suggesting a potential correlation between AI-2-mediated taxonomic variation and elevated substrate degradation capacity. In summary, the impact of AI-2 on *in vitro* rumen bacterial communities is not fixed, but exhibits clear substrate dependence. This study integrates community ordination, differential biomarker identification, co-occurrence network topology, functional prediction and enzyme activity quantification to provide new mechanistic evidence for understanding the role of quorum sensing signals in regulating *in vitro* bacterial community succession, and offers a theoretical basis for improving the utilization of ruminant roughage based on signal-mediated regulation strategies targeting fibrous substrate fermentation.

## Data Availability

The raw sequencing reads of 16SrRNA sequencing are available in the CNCB (https://ngdc.cncb.ac.cn) under BioProject accession number PRJCA061917.

## Footnotes

1
https://docs.qiime2.org/2022.11/tutorials/

2
https://metacyc.org/

3
https://www.kegg.jp/
