# Supplementary material for: Effects of Autoinducer-2 on rumen bacterial community succession during in vitro fermentation with oat and Suaeda salsa straws
Source: Front Microbiol. 2026 Aug 5;17:1880916. doi: 10.3389/fmicb.2026.1880916 (PMC13486247; doi:10.3389/fmicb.2026.1880916)
Supplement: Supplementary file 1 [file Data_Sheet_1.PDF]

Experimental workflow of anaerobic serial passaging culture of rumen microorganisms

Straw substrate pretreatment, anaerobic culture construction, serial passaging, and 16S rRNA gene sequencing

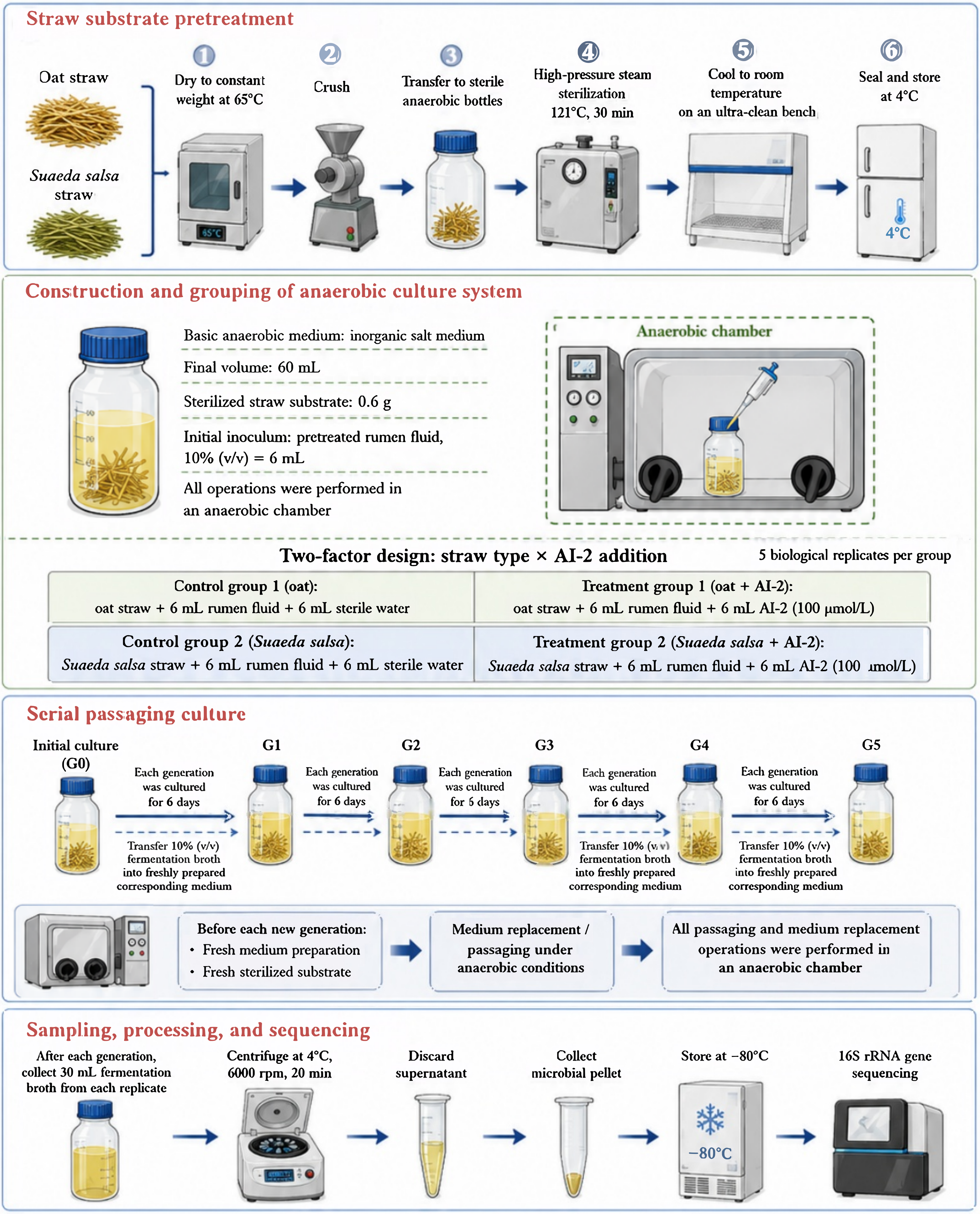

Samples selected for 16S rRNA gene sequencing

G0: A (original rumen fluid)	G1: Byck, ByAI_2, Bjck, BjAI_2	G3: Cyck, CyAI_2, Cjck, CjAI_2	G5: Dyck, DyAI_2, Djck, DjAI_2
